# Species occurrence of ticks in South America, and interactions with biotic and abiotic traits

**DOI:** 10.1038/s41597-019-0314-0

**Published:** 2019-12-03

**Authors:** Agustin Estrada-Peña, Santiago Nava, Evelina Tarragona, Sergio Bermúdez, José de la Fuente, Ana Domingos, Marcelo Labruna, Juan Mosqueda, Octavio Merino, Matias Szabó, Jose M. Venzal, Alberto A. Guglielmone

**Affiliations:** 1 Department of Animal Pathology, Faculty of Veterinary Medicine, Zaragoza, Spain; 2 INTA, Rafaela, Santa Fe, Argentina; 3 Instituto Conmemorativo Gorgas de Estudios de la Salud, Panama City, Panama; 4 SaBio, Instituto de Investigación en Recursos Cinegéticos (IREC-CSIC-UCLM-JCCM), Ronda de Toledo s/n, 13005 Ciudad Real, Spain; 5 Department of Veterinary Pathobiology, Center for Veterinary Health Sciences, Oklahoma State University, Stillwater, OK 74078 USA; 6 Global Healyh and Tropical Medicine, Instituto de Higiene e Medicina Tropical, Universidade Nova de Lisboa, Lisboa, Portugal; 7 Universidade de Sao Paulo, Sao Paulo, Brazil; 8 Universidad Autónoma de Querétaro, Querétaro, México; 9 Universidade de Tamaulipas, Tamaulipas, México; 10 Universidade Federal de Überlandia, Überlandia, MG Brazil; 11 Universidad de la República, Salto, Uruguay

**Keywords:** Community ecology, Biogeography

## Abstract

The datasets of records of the distribution of ticks and their hosts are invaluable tools to understand the phylogenetic patterns of evolution of ticks and the abiotic traits to which they are associated. Such datasets require an exhaustive collection of bibliographical references. In most cases, it is necessary the confirmation of reliable identification of ticks, together with an update of the scientific names of the vertebrate hosts. These data are not easily available, because many records were published in the so-called “grey literature”. Herein, we introduced the Dataset of Ticks in South America, a repository that collates data on 4,764 records of ticks (4,124 geo-referenced) with a special reference to an extra 2,370 records of ticks on cattle, together with a set of abiotic traits, curated from satellite-derived information over the complete target region. The dataset includes details of the phylogenetic relationships of the species of hosts, providing researchers with both biotic and abiotic traits that drive the distribution and evolution of ticks in South America.

## Abstract (summary)

**Table:** 

Measurement(s)	climate • tick infestation • Host-Parasite Relationship • phylogenetic data
Technology Type(s)	satellite imaging • digital curation
Factor Type(s)	time • species of vertebrate host
Sample Characteristic - Organism	Ixodidae
Sample Characteristic - Environment	terrestrial environmental zone
Sample Characteristic - Location	South America • Neotropics

Machine-accessible metadata file describing the reported data: 10.6084/m9.figshare.10108703

## Background & Summary

The ixodid ticks of South America are a clade of species that show taxonomic affinities with species reported to occur in the Tropical and the Palearctic-Nearctic fauna, with unique host-tick adaptations to the local vertebrate fauna^1^. These relationships could be considered a consequence of the separation of land masses between Africa and South America in geological times and the invasive events that took place between the Nearctic and the Neotropical regions^2^. Information about this large assemblage of species of the family Ixodidae, their biotic (hosts) and abiotic (climate) associations has been largely ignored at a continental scale because of the lack of an integrative dataset. Regional studies, or analyses including taxonomically close clades of species exist, linking intensive sampling in the field with molecular features of populations and the abiotic traits delineating these populations^3–5^.

We previously presented a novel framework that escalates from environmental traits to the relationships of a community of ticks and vertebrate hosts^6^. In this study we aimed to formulate a synthesis of the relationships linking the three corners of the triangle that supports the circulation of tick-transmitted pathogens in South America: the vertebrate hosts, the ticks, and the environmental traits. We also summarized the reported distribution and abiotic relationships of the ticks recorded on domestic cattle, which is a growing economic problem in the region. The synthesis presented in this study addresses the consolidation of the community of ticks and the factors governing it. Commonly, the relationships between ticks and their hosts have been addressed under the biotic (hosts) or abiotic (environmental) perspectives of factors governing specialization and distribution of ticks. Previous studies provided datasets of the distribution of tick parasites of livestock in the region^7,8^, revisions of the ecology of some species^9,10^, taxonomic guides for tick classification^11^, descriptions of poorly known species, or efforts addressed to understand the consequence of the climate trends on the tick parasites of cattle^12–16^. Previous reports approached the taxonomic aspects of the ticks of the family Ixodidae in the region by addressing previous misconceptions about its systematics^1,3–5^. A complete dataset of environmental traits, *bona fide* records of ticks, accurate coordinates of the distributional data, and a complete and updated list of hosts, together with the complete set of literature references, is the requirement for a community approach to the study of the ixodid ticks in South America.

In this context, we provide the necessary tools for prospective studies on distributional mapping, phenology of ticks with economic interest, species environmental modeling, and relationships among species of ticks and hosts. A list of 880 references sets the literature basement for further research on this topic. This dataset is thus focused on the ecological and environmental research, including data of the phylogenetic relationships of wild hosts to enable a wider research on a set of parasites that has been only rarely approached from the perspective of the community^17,18^ or predictive mapping^12–16^.

## Methods

This dataset consists of four elements, namely (i) the summarized climate for the period 2002–2018 obtained at a resolution of 0.05° from the MODIS-Terra series of satellites, as monthly averaged values of the Land Surface Temperature (LSTD) and the Normalized Difference Vegetation Index (NDVI), (ii) the data of the distribution (with coordinates) of ticks parasites of wild animals in South America, and (iii) the data of the distribution of ticks affecting cattle in the Neotropics (with coordinates). Both parts (ii) and (iii) are complemented by the list of references on which they are based. A complimentary part (iv) includes data on the phylogenetic relationships of vertebrate hosts, and the relationships of each species of tick with that phylogenetic tree. Part (i) is intended to provide an abiotic background for future studies on the ecology of the ticks in South America, together with already existing datasets^19^ of the bioclimatic regions of South America. The item (ii) constitutes the largest compiled dataset on the distribution of ticks in South America that are reported on wild vertebrates, volant and terrestrial. Part (iii) is intended as a dataset of the reported distribution of ticks affecting domestic cattle, which are considered a serious constraint in animal production. Part (iv) aims to complement the previous sections, providing data of the phylogenetic tree of the wild hosts and thus the relationships of the species of ticks with the vertebrate fauna in South America. Thus, the set of records for each species of tick can be unambiguously tracked back to both its biotic (hosts) and abiotic (climate) components. This results in the better available picture of the distribution of ticks in South America, aiming to jointly display both the biotic and abiotic constraints of their distribution.

### Climate data

The purpose of including climate data in the main dataset of the distribution of ticks in South America is the production of a coherent set of data that describes the environmental traits that shape the environmental volume of the tick, the Grinellian niche^19^. We used remotely sensed information on temperature and vegetation, producing the average monthly values of both LSTD and NDVI. The latter is a measure of photosynthetic activity, which has been used as a proxy for vegetation stress, an important feature affecting the survival of ticks.

The raw LSTD and NDVI data were obtained from the MODIS website (https://lpdaac.usgs.gov/product_search/; last accessed, January 2019). at monthly intervals for the years 2002–2018, with a spatial resolution of 0.05°. The products used were MOD13C2.006 and MOD11C3.006, accessed and downloaded using the package MODIStsp^20^ for the R programming environment interfacing the MODIS API. After calculation of the monthly averaged values for LSTD and NDVI over the period 2002–2018, we produced one complete year at monthly intervals that reflects the average values of either LSTD or NDVI in the target region. Monthly averages were obtained for the complete 2002–2018 period using the flags issued by the MODIS team about pixels contaminated by ice, cloud or snow. The pixels marked as “unreliable” were excluded from calculations to obtain an adequate estimate of the monthly average. This is why some areas in the territory have no values, because they are permanently covered by clouds, ice, or are located in areas such as the Andean range or the extreme Southern. The region of interest covers the coordinates 34°N, 120°W, 57°S, 33°W. This includes areas of Northern South America, but we aimed to produce information for nearby regions because some species can extend their ranges out of the target area. All the images are projected in WGS84 (Latitude-Longitude) [Data record 1].

We carried out an unsupervised classification of the region, using the monthly averaged values of LSTD and NDVI. The purpose of the unsupervised classification is to produce a summarized background of the abiotic traits over the complete territory, as “categories of climate” that are independent but complement existing classifications of the region (https://www.natureserve.org/conservation-tools/ecological-systems-latin-america-and-caribbean/; last accessed, January 2019). Existing classifications commonly assign the name of the biome based on expert classification but omit an explicit and quantitative background of the environmental traits observed in one complete (average) year. Each different group of pixels belonging to a “category” has been associated to the monthly values of LSTD and NDVI recorded for the patch producing a database of ecological categories of the habitat in the target region. The dataset is projected in WGS84 (Latitude-Longitude) [Data record 2].

### Data of the distribution of ticks

The dataset including the records of the distribution of ticks in South America was obtained from a wide variety of sources. The method for collecting tick occurrences was based on a critical review of tick literature, encompassing Neotropical tick studies for a period of about 130 years. This effort exceeded current protocols for literature search because the need to track many studies included in “gray literature” or in journals that no longer exist but found to contain reliable information of local or regional importance. One of the co-authors (AAG) performed the first perusal of the papers to exclude studies with ambiguous information and updating historical information to current taxonomical tick knowledge. The update of the scientific names of hosts was carried out by another co-author (AEP). Thereafter the information was shared with the authors to further discuss controversial records to finally construct the current database that reflects the scenario of Neotropical tick ranges. The valid scientific names of tick species were based on available data (http://rafaela.inta.gob.ar/nombresgarrapatas/). The valid scientific names of vertebrates were based on the list available at GBIF (http://www.gbif.org). Modern literature was mainly queried through Internet on journals searchable in Thomson Reuters, Scopus, and PubMed. The search of data in indexed journals was done primarily by AAG, with support by SN and ET (see the list of author’s contributions) with contributions by the other co-authors. The query used only the scientific names of the genera of ticks as the keywords, together (AND) with the names of the countries in the region. We did not include any additional terms to the query to obtain as many papers as possible, even if not related completely with the topic of the dataset. A total of 2377 papers were obtained, which were examined visually by AAG (title and abstract) to exclude those without explicit information about tick distribution, species or hosts of the family Ixodidae (i.e. describing treatments without geographical information, studies on laboratory specimens, etc.). A ‘record’ is thus a dual combination of a tick and a species of host at one site. If the same combination of partners was collected at the same site several times (e.g., through seasonal collections), it was included only once. However, if different references reported the same association in the same place, all of them were included, looking for completion of the references.

The compilation of the distribution of ticks in South America parasites of wild vertebrates produced information about 74 species of ticks of the genera *Amblyomma*, *Ixodes*, and *Haemaphysalis*, with a total of 4,764 unique records [Data record 3]. This large dataset includes information on the species of ticks, hosts, localities and country of collection, the coordinates (if known), and the citing reference. The same file is provided in a GIS open format, projected in WGS84 (Latitude-Longitude) [Data record 4]. If not explicitly mentioned in the paper, coordinates were obtained from local gazetteers and with the expert opinion of the co-authors about the region of origin.

The co-authors agreed on a list of species that affect domestic cattle in the Neotropics, consisting of 1 species of the genus *Dermacentor*, 2 species of the genus *Rhipicephalus*, 11 species of the genus *Amblyomma*, 1 species of the genus *Haemaphysalis*, and 3 species of the genus *Ixodes*. Data were obtained from the same bibliographical sources as above, but also from the personal records of the co-authors [Data record 5]. This dataset has been converted to a point shape file, designed for GIS purposes and projected in WGS84 (Latitude-Longitude) [Data record 6]. The complete list of references is also provided in a “.rtf” file as part of this dataset [Data record 7] containing 880 references between the years 1880 and 2019.

### Phylogenetic relationships of hosts

We aimed to provide a background allowing evaluation of the relationships between the ticks and the phylogenetic diversity of the vertebrate hosts. To obtain the phylogenetic relationships of the vertebrates, we queried the Open Tree of Life (OTL, http://www.opentreeoflife.org/; accessed February 1st, 2019) using the package “rotl”^21^ for R. We found phylogenetic information for 206 genera of vertebrates in South America, reported as hosts of the ticks; the use of species of hosts to build the phylogenetic tree produced an over-dispersion of records and decreased the information. The purpose of this part of the study is to provide complementary information about the hosts used by each species of ticks, separately for larvae, nymphs and adults, because some species may use different groups of hosts according to the developmental stage. We obtained the file in nexus format from OTL, on which we plotted the relationships among ticks and hosts. To avoid a bias of results, plots of relationships among ticks and hosts were produced only for species and stages with more than 15 records (even without coordinates). We produced these plots using the package “phyloclim”^22^ for R [Data record 8].

## Data Records

The data obtained and curated as explained above, are documented in Table 1 and available in DataDryad^23^. This manuscript adheres to the FAIR principles^24^ since all the data are stored in a public repository, are findable on the Internet, and can be manipulated and/or updated.

**Table 1:** Summary of the complete dataset generated and described in this study. The table indicates the name of each file in the dataset, the structure of the files, and the main authors involved in its preparation.

	Description	Structure of the files	Main author and co-authors
Data report 1	Climate dataset	Two sets of files: 24 files (format “.img”) that contain monthly values of the ground temperature (LSTD, in Kelvin) and the Normalized Difference Vegetation Index (NDVI, unit less). Projection: WGS84	AEP
Data report 2	Categories of climate	A shapefile with 12 columns on monthly values of the ground temperature in Kelvin (LSTD) and 12 columns with NDVI. Projection: WGS84	AEP
Data report 3	Records of ticks on wild hosts	Excel type file (“.xlsx”) including information of the records of ticks on wild vertebrates, including species of tick and host, coordinates of the point of collection, and publication of reference.	AAG, SN, ET
Data report 4	A GIS file in shape file format (“.shp”)	A shapefile with the records of ticks on wild hosts. Projection: WGS84	AEP, AAG, SN, ET
Data report 5	Records of ticks on cattle	Excel type file (“.xlsx”) including information of the records of ticks on cattle, coordinates of the point of collection, and publication of reference	AAG, SN, ET, SB, with contributions by the rest of co-authors
Data report 6	A GIS file in shape file format (“.shp”)	A shapefile (“.shp”) containing the records of ticks in cattle. Projection: WGS84	
Data report 7	List of references	A “.rtf” file including the references of the published papers from which information was obtained	AAG, SN, ET
Data report 8	Phylogenetic tree of the genera of hosts with plots of the use of each host by each species of tick	A “.zip” file containing 126 files in “.pdf” format that display the phylogenetic relationships of the genera of hosts in South America and the relationships of each species and stage of the ticks with these hosts. Each blue dot means for reports of the stage/species on these hosts, its size is proportional to the number of reports.	AEP

## Technical Validation

### Climate data

The climate dataset of monthly averages of LSTD and NDVI was produced using the flags issued by the MODIS team indicating pixels contaminated by ice, clouds or snow. Using the software qGIS v3.4 (http://www.qgis.org, last accessed January 2019) we removed the contaminated pixels at every step before producing the average for each month. Once the monthly averaged values were obtained, we did check the statistics of each monthly layer, looking for abnormally high or low values. These issues were addressed individually looking for the source of the “wrong value” (i.e. long-lasting contamination of pixels by snow in the mountains) and were replaced with a “−9999” value.

### Literature search

It was evident at initial stages of the bibliographic search that the use of a restrictive set of keywords would miss papers that fit the criteria of the search. In other words, the submission of a search chain based on the scientific names of the ticks AND the scientific names of the possible hosts, AND every country of the target region would lack specific information for some species of ticks or hosts not previously recognized in the target territory. Therefore, we performed a deliberately relaxed query including only the scientific names of the genera of the ticks and countries, and to keep only the papers dealing with ecological information after a critical evaluation of the abstract. The reported tick records were assessed for reliability mainly by AAG (with support by SN and ET). It was necessary to check the reliability of the identification of each record, comparing with further publications and using expertise on the distribution of ticks in South America to produce the most reliable picture. The list of scientific names of vertebrates was updated by AEP, using data from the GBIF database (www.gbif.org; last accessed, January 2019). Geo-references were included “as is” in the case they were included in the original report. For erroneous coordinates, a correction was made looking for the name of the locality (if reported) and assigning the correct coordinates. If coordinates were not published in a given paper, a search in geo-referencing services was done, using also expertise on local names, regions, and countries, and carried out mainly by AAG (with support by SN and ET). We did not manage to geo-locate every single record, and data are entered for locality, country, species of tick and host. In some cases, the reliability of the identification or the name of the administrative divisions did not match. In some instances, the identification of the tick was considered to be unreliable, because the presumed record was far from the accepted range of distribution, or the association with the reported host was very different of the usual range of hosts of a species. All these details are included in an extra field of the dataset, in which unreliability of the identification and/or the geo-location, are mentioned.

This part of the literature search had a variable difficulty for assessment of quality and completeness since these communications include papers in local journals, internal reports of national institutions involved in animal health or wildlife ecology, and communications to congresses. They are rarely indexed but are the backbone of this dataset, thus providing researchers with a large number of records that are hidden to common searches on the Internet.

### Phylogenetic relationships of hosts

We did not approach a technical validation of the phylogenetic tree of hosts, since that verification is routinely carried out by the technical staff of Open Tree of Life. According to the web site (OTL, http://www.opentreeoflife.org/; accessed February 1st, 2019) quality control is performed with in-house algorithms to produce scientific level data.
